# Transcriptome Analysis of *Arabidopsis* GCR1 Mutant Reveals Its Roles in Stress, Hormones, Secondary Metabolism and Phosphate Starvation

**DOI:** 10.1371/journal.pone.0117819

**Published:** 2015-02-10

**Authors:** Navjyoti Chakraborty, Priyanka Sharma, Kostya Kanyuka, Ravi R. Pathak, Devapriya Choudhury, Richard A. Hooley, Nandula Raghuram

**Affiliations:** 1 University School of Biotechnology, G.G.S. Indraprastha University, Sector 16 C, Dwarka, New Delhi, 110078, India; 2 Dept. of Plant Biology and Crop Science, Rothamsted Research, West Common, Harpenden, Hertfordshire, AL5 2JQ, United Kingdom; 3 School of Biotechnology, Jawaharlal Nehru University, New Delhi, India; 4 Dept. of Biology and Biochemistry, University of Bath, Claverton Down, Bath, BA2 7AY, United Kingdom; University of Malaga-Consejo Superior de Investigaciones Científicas, SPAIN

## Abstract

The controversy over the existence or the need for G-protein coupled receptors (GPCRs) in plant G-protein signalling has overshadowed a more fundamental quest for the role of AtGCR1, the most studied and often considered the best candidate for GPCR in plants. Our whole transcriptome microarray analysis of the GCR1-knock-out mutant (*gcr1-5*) in *Arabidopsis thaliana* revealed 350 differentially expressed genes spanning all chromosomes. Many of them were hitherto unknown in the context of GCR1 or G-protein signalling, such as in phosphate starvation, storage compound and fatty acid biosynthesis, cell fate, etc. We also found some GCR1-responsive genes/processes that are reported to be regulated by heterotrimeric G-proteins, such as biotic and abiotic stress, hormone response and secondary metabolism. Thus, GCR1 could have G-protein-mediated as well as independent roles and regardless of whether it works as a GPCR, further analysis of the organism-wide role of GCR1 has a significance of its own.

## Introduction

G-protein signalling pathways are implicated in a variety of plant processes, but their upstream receptors and the role of typical G-protein coupled receptors (GPCRs) in plants are not very well understood [1,2]. The canonical GPCR gene in *Arabidopsis* (GCR1) was isolated independently by two groups [3,4]. The latter group initially called it a cytokinin receptor but subsequently found that cytokinin response was due to an independent mutation [5]. Others implicated GCR1 in PIPLC mediated regulation of DNA synthesis [6], abolishing seed dormancy and reducing flowering time [7]. Later, knock-out mutants of GCR1 were used to implicate it in seed germination in response to brassinosteroids and gibberellins [8], reducing drought stress, negative regulation of ABA induced gene expression, ABA and S1P-induced regulation of stomatal aperture [9], blue light response [10] etc. Additional GPCR candidates were reported based on their transmembrane domains [11–13], with the list eventually swelling upto 56 candidates, of which, GCR1 is bioinformatically considered to be the best candidate based on GPCR fold analysis [14]. Another candidate, GCR2, which was reported to be a 7TM ABA receptor [15] was later reported to be a homolog of lanthionine synthetase [16,17]; and eventually found to be a lantibiotic cyclase like protein with no role as a GPCR [18].

Conventional GPCRs act as ligand-activated guanine nucleotide exchange factors (GEF) to release GDP from the G-protein alpha subunit. Despite being the most studied candidate, the role of GCR1 as a GPCR remains biochemically unproven, due the lack of an identified ligand that binds GCR1, the lack of demonstration of GEF activity and the disagreement over its reported [9,12] physical interaction with G-protein alpha subunit, GPA1 [19,20].

Another argument against the role of GPCRs in plant G-protein signalling was based on the finding that the rate limiting step in plant G-protein signalling is the acceleration of GTP hydrolysis by another 7TM protein coded by AtRGS1, a GTPase accelerating/activating protein, and not GEF activity of GPCR [21,22]. This led to the assertions that plants neither have GPCRs nor require them for G-protein signalling, and that plant GPCRs predicted so far are mostly mis-annotated [23]. Another reasoning against plant GPCRs was that their homologs are also found in green algae that lack other components of G-protein signalling [24], but a complete G-protein complex was recently found in the green alga *Chara braunii* [25]. This, coupled with the lack of GAP/RGS proteins in grasses [22] and the recent report that a single pass leucine rich repeat (LRR) receptor-like protein acts like a GPCR in maize, with homologs abundant in the plant kingdom [26] point to other possibilities in receptor-G-protein coupling that cannot be completely ruled out yet. Even in the case of GCR1, the strongest GPCR candidate, there is no reason to give up hopes of finding a GCR1 ligand or GEF activity, as there is no reported evidence of failure so far.

Thus, while the controversy over the existence or requirement for G-protein coupled receptors (GPCRs) in plant G-protein signalling is far from settled, we have reframed the question, as to what is the overall role of GCR1 in *Arabidopsis*, rather than whether it codes for a GPCR or not. Functional genomics from a gene discovery perspective allows fundamental investigations into the genomewide role of GCR1 in *Arabidopsis*, including, but not limited to its role in G-protein-regulated processes. It may even contribute to resolving the current controversy to some extent. In this paper, we used whole transcriptome microarray analysis of a GCR1 knock-out mutant and its wild type in *Arabidopsis* for the first time to identify some important genes, pathways and responses regulated by GCR1, some of which, (but not all), are also regulated by G-proteins.

## Materials and Methods

### Isolation of GCR1 mutant

A T-DNA tagged mutant population from the *Arabidopsis* Knockout Facility at the University of Wisconsin [27] was screened by PCR for disruption of *GCR1* gene: The population consisted of 72,960 BASTA (glufosinate)-resistant lines of *Arabidopsis thaliana* ecotype Ws2 transformed with an activation-Tag vector pSK1015 [28]. The GCR1 mutant was detected by DNA gel blot analysis of PCR-amplified products in DNA super-pool 40 of the BASTA population using combinations of GCR1-specific primers KK66 [located upstream of the ATG start codon of GCR1 ORF] (5′-AAATCGTCAATTCAATCTCTCAGATCAGT-3′) or KK68 [locates downstream of the TGA stop codon of GCR1 ORF] (5′-GCGCCGGTTTAAGTGATAGTATTTTCATA-3′) with the left T-DNA border specific primer JL202 (5′-CATTTTATAATAACGCTGCGGACATCTAC-3′). The PCR reaction contained 1X Takara Ex-Taq polymerase buffer (Takara), 0.2 mM deoxynucleotide triphosphates (dNTPs), 0.24 pmol/μl gene-specific primer, 0.24 pmol/μl JL202 primer, and 0.05 unit/μl Takara Ex-Taq polymerase. The PCR conditions were 96°C for 5 min and 36 cycles at 94°C for 15 s, 65°C for 30 s, and 72°C for 2 min each. Sequencing of KK66-JL202 and KK68-JL202 PCR products revealed multiple T-DNA integration.

Seeds corresponding to the identified super-pools and sub-pools were grown and DNA was extracted from leaves for genotyping and sequencing of the mutant lesions. The primers KK66 and KK68 were used to detect the *GCR1* wild-type copy, and primers KK66 and JL202 were used to detect the presence of the T-DNA in the *GCR1* gene. PCR conditions were as above. PCR products were separated on agarose gels, and individual segregating plants for the GCR1 mutant (*gcr1–5*) were genotyped based on the presence or absence of wild-type and T-DNA bands.

### Phenotypic characterization of the mutants

Seeds of the wild type, Ws2 and GCR1 mutant (*gcr1–5*) were surface sterilized using 70% ethanol and washed thrice with sterile ultrapure water and stratified at 4°C for two days on half-strength B5 plates. The plates were then kept in growth chamber maintained at 22±1°C with a light intensity of 150 μM sec^-1^ m^-2^ and a photoperiod of 16:8 (light:dark). 10 day old plantlets were then transferred to 3.5 cm pots containing 1:1 mixture of soilrite and vermiculite. The pots were watered using sub-irrigation. The plants were allowed to grow for full life cycle and various phenotypic characters were measured at appropriate durations.

### Plant material and RNA isolation

*Arabidopsis thaliana* GCR1 mutant (*gcr1–5*) as well as the corresponding wild type, were grown on 1X B5 medium hydroponically in a growth chamber at 22±1°C with a light intensity of 150 μM sec^-1^ m^-2^ and a photoperiod of 16:8 hours of light:dark cycle. The seeds were vernalized prior to inoculation at 4°C for 2–3 days. Total RNA was isolated from 3–4 week old whole seedlings using a modified hot phenol-LiCl method optimized on *Arabidopsis* [29]and reported in the context of cyanobacteria [30]. RNA samples were analyzed by Nanodrop spectrophotometer and Bioanalyzer (Agilent technologies, Santa Clara, USA) to determine the quality, quantity and suitability for microarray. RNA samples having a RIN (RNA Integrity Number) value greater than 6.0 were used for microarray experiments. The isolated RNAs were also used for validating the mutants using qPCR with gene-specific primers.

### Microarray experiments and data processing

Microarray experiments were performed using Agilent 8×60k *Arabidopsis* array (AMADID 037661) with independent biological duplicates both the wild type, Ws2 and GCR1 mutant. Total RNA was transcribed into Cy3 labelled cRNA using Agilent Quick-Amp labelling kit as per manufacturer’s instructions. Labelled cRNA was purified using RNeasy minikit (Qiagen) and the specific activity of cRNA was determined as a quality control for all the samples. They were hybridized with the microarrays using Agilent *in-situ* hybridization kit as per manufacturer’s instructions. The washed slides were scanned and the images were manually verified to ensure that they are devoid of uneven hybridization, streaks, blobs and other artifacts. Hybridization across the slide was analyzed based on the number of features that were positive and significantly above background, i.e g(r) is PosAndSignif. Overall the microarray images were clean, had uniform intensity and with very low background noise. The data was then extracted from images by using Feature Extraction 10.7 software (Agilent Technologies).

### Data analysis

The data was then normalized using the recommended ‘Per Chip and Per Gene Normalization’ feature of the software GeneSpring GX Version 11.5. The correlation of replicates was checked using principal component analysis and correlation coefficients were obtained. The geometric mean (geomean) fold change values are represented as log2. The average data of biological replicates was taken for final calculations. Log2fold change value of 1.0 with p-value of 0.05 was taken cut-off for differential-regulation.

### Functional classification of significant genes

The differentially regulated gene lists were assigned gene ontology terms according the *Arabidopsis* Information Resource (TAIR 10) [31]. The differentially regulated gene lists were subjected to enriched GO categorization using AgriGO with default settings. Pathway analysis of the DEGs to obtain the list of changed pathways was done using plant MetGenMAP, which takes AraCyc as the background. Further functional classification was also carried out using Mapman tool, where the DEGs were assigned to different biological processes (bins). This tool also takes into account the log2fold change and represents it as coloured boxes on the software generated biological process map.

### Data validation using qPCR

Differentially expressed genes obtained from microarray analyses were verified by RT-qPCR using Stratagene Mx3000P (Agilent technologies). Typically, total RNA was digested by RNase-free DNase (Fermantas), repurified, quantified and 5 μg of RNA was used for cDNA preparation for each biological replicate using Oligo(dT) primers and RevertAid reverse transcriptase (Fermentas). The analyses were done using biological triplicates, out of which two were the same as used for microarray. Sequences for designing the primers were obtained from TAIR. PCR amplifications were performed in 20 μl by using the BrilliantIII Ultrafast SYBR Green QPCR mastermix (Agilent Technologies) with 1.0 μl of sample cDNA and 100 nmoles of each gene-specific primer. Primer efficiency was determined by serial dilution of the template and only primers that worked at 90–110% efficiency were used for all qPCR analyses. The specificity of primer pairs was obtained by melting curve analysis of the amplicons. Actin2 (ACT2) was used as an internal control for normalization. Quantification of the relative changes in gene expression was performed by using Pffafl method [32].

## Results

### GCR1 mutant characterization

**Fig 1:**
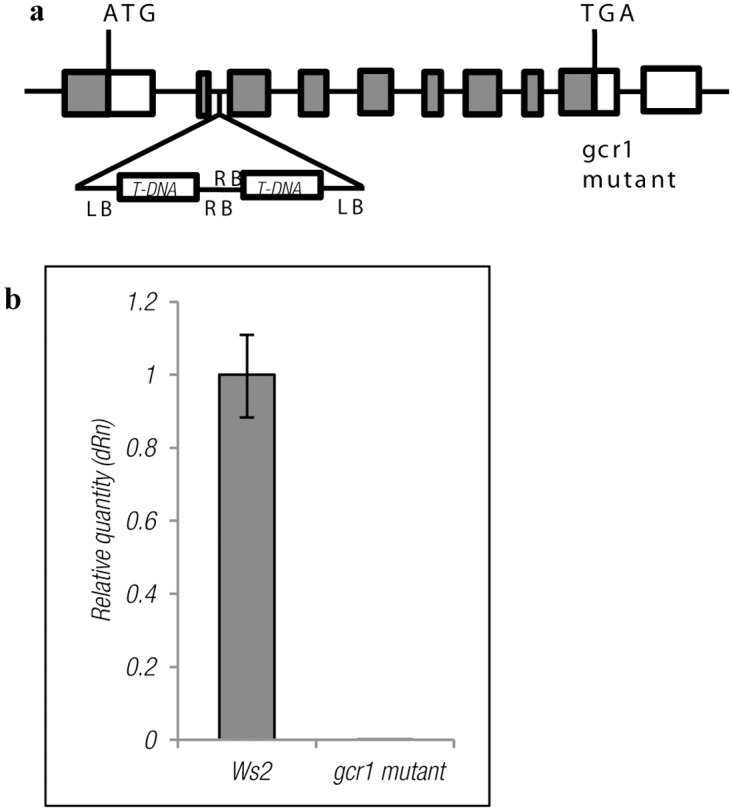
(a) T-DNA insertion site/orientation in the mutated gene of GCR1. The exons are represented as boxes and the introns are represented as lines. LB and RB represent the left and right border respectively. (b) qPCR validation of the mutant. The real time RT-PCR was performed in triplicate using independent samples of total RNA and the values are represented as relative quantity ± SE.

Sequencing of the PCR-amplified GCR1 gene confirmed the mutation in the second intron of GCR1 (Fig. 1A), similar to the mutant *gcr1–3* described previously, except that it had two T-DNAs with their right borders facing each other, instead of the single T-DNA insertion reported earlier [9]. qPCR with gene-specific primers showed no expression of GCR1 transcript, confirming that this is a knock-out mutation (Fig. 1B). The mutant plants (*gcr1–5*) were phenotypically characterized for root length, plant height, leaf shape, etc. It was found that other than total number of siliques (S1 Fig.), *gcr1–5* is barely distinguishable from the wild type as is the case with other known GCR1 mutants [8,9].

### Microarray analysis and validation

**Fig 2:**
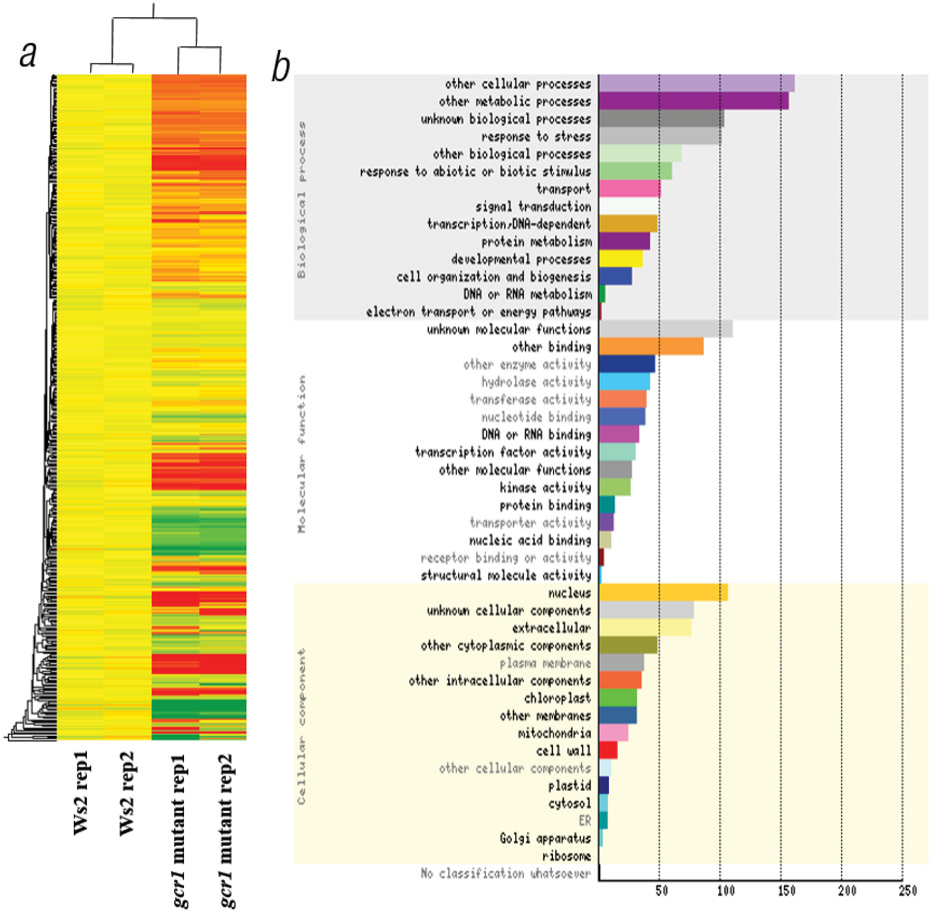
(a) Heat map of differentially expressed genes. The background-subtracted microarray data was subjected to hierarchical clustering using Genespring software ver. 11.5 to generate the heatmap. Yellow represents the control data, while red and green represent up-regulation and down regulation respectively. (b) GO categorization of DEGs. The DEGs were categorized into GO classes using classification superviewer tool of Bioarray resource (www.bar.utoronto.ca)

**Fig 3:**
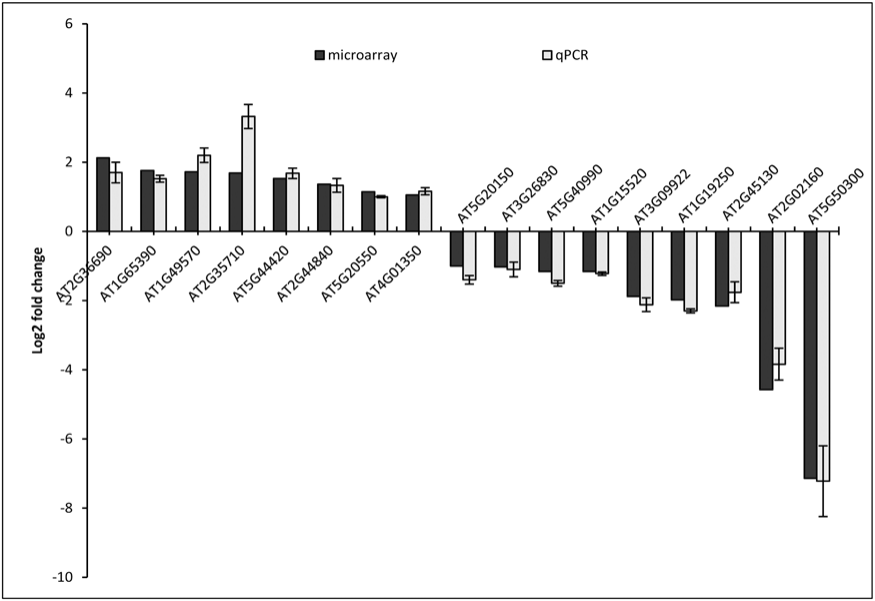
Validation and comparison of microarray results using qPCR for a few genes selected from each of the important biological processes. The experiment was carried out using biological triplicates and the values are presented as log2FC ± SE. (AT2G36690–2-OG; AT1G65390—ATPP2-A5; AT1G49570—peroxidase family protein; AT2G35710—PGSIP7; AT5G44420—PDF1.2; AT2G44840—ERF13; AT5G20550–2-oxoglutarate; AT4G01350—Cysteine/Hisidine-rich C1 domain family protein; AT5G20150—SPX1; AT3G26830—PAD3; AT5G40990—GLIP1; AT1G15520—PDR12; AT3G09922—IPS1; AT1G19250—FMO1; AT2G45130—SPX3; AT2G02160—CCCH type zinc finger family protein; AT5G50300—AZG2.

The experiment was carried out in accordance with MIAME compliance and the high correlation coefficients between biological replicates (>0.95) clearly indicate the robustness and a high level of reproducibility of the data (S1 Table). A stringent cut-off value of 1.0 (geometric mean log_2_) with a p-value of ≤ 0.05 was used for determining the up- or down-regulated genes in the mutant with respect to the wild type control. A total of 432 differentially regulated spots were obtained in the mutant (314 up-regulated and 118 down-regulated). These spots corresponded to 350 unique differentially expressed genes (DEGs) in the mutant (265 up-regulated and 85 down- regulated). A list of the top 20 up- and down-regulated genes are shown in Table 1. A heatmap of all the differentially regulated genes is shown in Fig. 2a. A set of 17 (8 up-regulated and 9 down regulated) genes representing all the affected biological processes were subjected to RT-qPCR using gene specific primers checked for efficiency (90–100%). The results of RT-qPCR matched with the microarray data in all the cases with a Pearson product moment correlation of 0.98 (p-value > 0.0001) (Fig. 3), validating the basic trends of regulation of gene expression on the microarray. The list of candidate genes along with the primer sequences are given in the S2 Table.

**Table 1:** List of the top 20 up-regulated and the top 20 down-regulated genes in the GCR1 mutant.

Gene Description	Locus id	Accession id	Gene name	Log2FC	p-value
Up-regulated in *gcr1–5*					
LOB domain-containing protein 27	AT3G47870	NM_114657	LBD27	8.07	0.0116
Paired amphipathic helix (PAH2) superfamily protein	AT1G24200	NM_102266	AT1G24200	5.47	0.0182
phospholipase-like protein (PEARLI 4) family	AT5G11140	NM_121152	AT5G11140	4.57	0.0002
Redox responsive transcription factor 1	AT4G34410	NM_119606	RRTF1	3.81	0.0166
Pathogenesis-related gene 1	AT2G14610	NM_127025	PR1	3.62	0.0173
Basic-leucine zipper (bZIP) transcription factor family protein	AT5G42910	NM_123656	AT5G42910	3.58	0.0348
other RNA	AT2G06002	NR_022465	AT2G06002	3.48	0.0030
C2H2 and C2HC zinc fingers superfamily protein	AT3G01030	NM_110968	AT3G01030	3.42	0.0118
aspartyl protease family protein	AT5G48430	NM_124218	AT5G48430	3.30	0.0187
S-locus lectin protein kinase family protein	AT1G11340	NM_101007	AT1G11340	3.25	0.0154
unknown protein	AT3G60647	NM_001125395	AT3G60647	3.23	0.0055
XH domain-containing protein	AT1G80970	NM_106745	AT1G80970	3.23	0.0363
Poltergeist	AT2G46920	NM_180132	POL	3.23	0.0211
Peroxidase 4	AT1G14540	NM_101321	PER4	3.15	0.0024
unknown protein	AT5G66810	NM_126079	AT5G66810	3.11	0.0289
unknown protein	AT3G55570	NM_115414	AT3G55570	3.10	0.0235
Cytochrome p450, family 94, subfamily b, polypeptide 3	AT3G48520	NM_114710	CYP94B3	3.01	0.0110
Ethylene response sensor 2	AT1G04310	NM_100312	ERS2	2.91	0.0420
Chromomethylase 1	AT1G80740	NM_106722	CMT1	2.87	0.0284
Unknown protein	AT3G28870	NM_113808	AT3G28870	2.87	0.0060
	Down-regulated in *gcr1–5*				
AZA-guanine resistant2	AT5G50300	NM_124409	AZG2	-7.14	0.0104
tRNA synthetase-related	AT5G10880	NM_121126	AT5G10880	-6.52	0.0049
Protein of unknown function	AT1G04890	NM_100367	AT1G04890	-6.34	0.0069
Homeodomain-like superfamily protein	AT5G62110	NM_125604	AT5G62110	-5.82	0.0063
Leucine-rich repeat protein kinase family protein	AT5G24100	NM_122315	AT5G24100	-5.26	0.0123
unknown protein	AT2G13547	EF183317	AT2G13547	-5.10	0.0016
unknown protein	AT5G24250	NM_122331	AT5G24250	-5.01	0.0402
CCCH-type zinc finger family protein	AT2G02160	NM_126276	AT2G02160	-4.57	0.0499
F-box associated ubiquitination effector family protein	AT3G06280	NM_111503	AT3G06280	-4.48	0.0350
Methionine sulfoxide reductase b7	AT4G21830	CD530941	AT4G21830	-4.34	0.0277
Late embryogenesis abundant (LEA) protein-related	AT5G54370	NM_124817	AT5G54370	-4.08	0.0143
Potential natural antisense gene	AT1G71828	NR_027728	AT1G71828	-3.96	0.0391
unknown protein	AT5G35870	NM_122978	AT5G35870	-3.95	0.0019
unknown protein	AT5G03440	NM_120423	AT5G03440	-3.80	0.0082
Similar to yeast POP1	AT2G47300	NM_001084603	AT2G47300	-3.74	0.0208
Proline-rich extensin-like family protein	AT1G26250	NM_102389	AT1G26250	-3.67	0.0041
unknown protein	AT3G32896	NM_148781	AT3G32896	-3.66	0.0199
unknown protein	AT3G50674	NM_001125338	AT3G50674	-3.66	0.0236
Leucine-rich repeat protein kinase family protein	AT1G24650	NM_102307	AT1G24650	-3.49	0.0363
Similar to TSK-associating protein 1	AT3G15950	NM_112465	NAI2	-3.44	0.0077

### Stress response genes among GCR1-regulated genes

**Fig 4:**
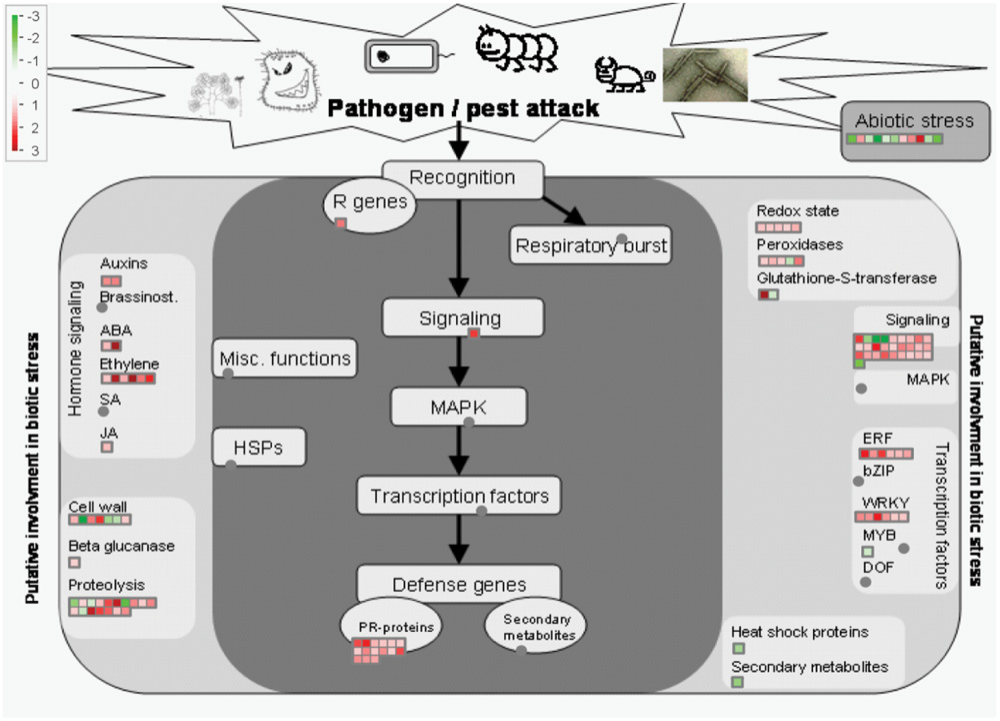
Mapman analysis of genes differentially regulated in *gcr1* mutant. Out of the total list of 350 DEGs, 119 mapped onto biotic stress response. The red dots represent the up-regulated genes, green dots represent the down-regulated genes and the grey dots represent the genes to which none of the DEGs were assigned. The level of differential regulation is according to the scale given.

**Fig 5:**
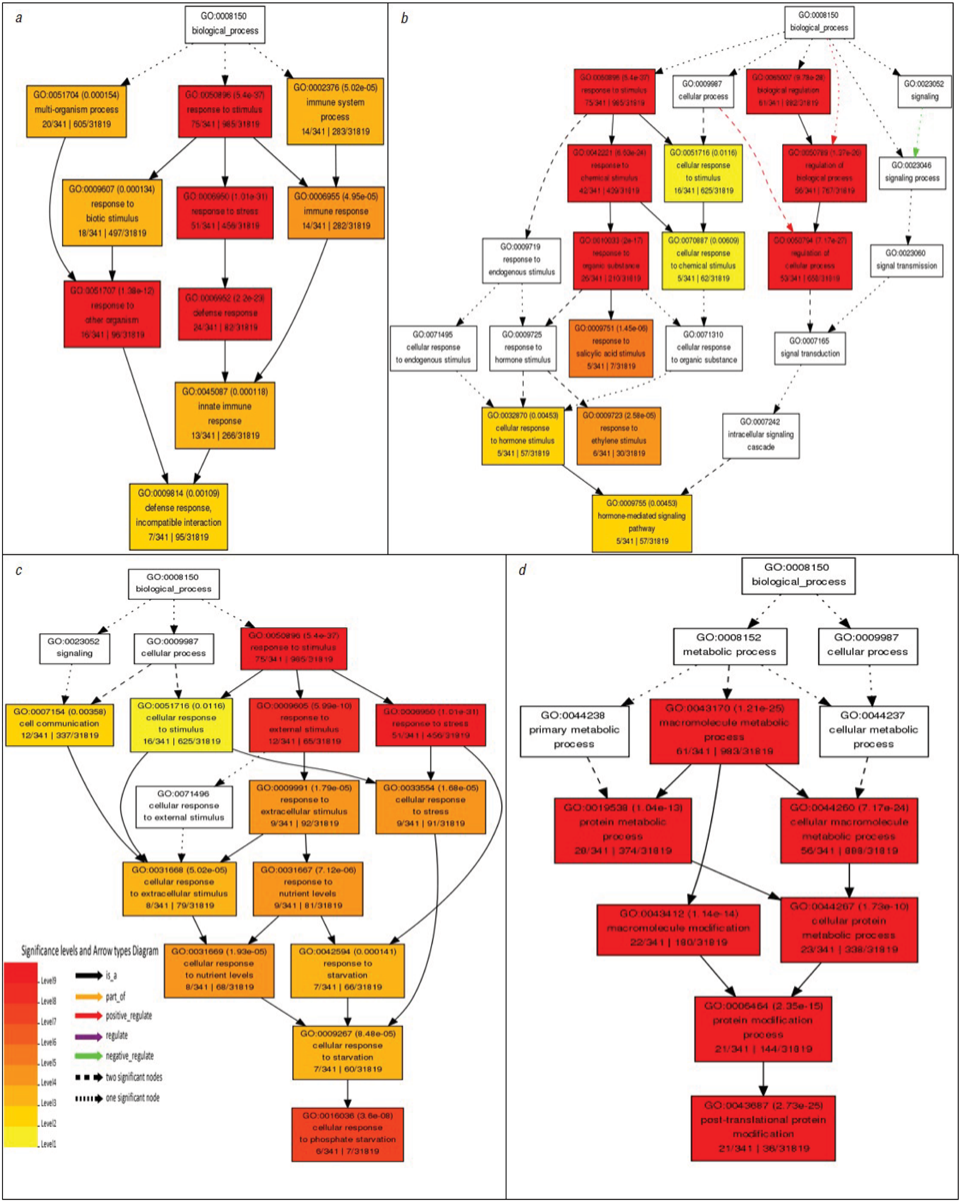
Singular enrichment analysis (SEA) of the DEGs using AgriGO into important biological processes. (a) Stress response (b) Hormone response (c) Response to Phosphate starvation (d) Protein modification.

Mapman analysis mapped 119 DEGs as belonging to the biotic stress response category (Fig. 4), including some genes that are involved in both biotic and abiotic stresses and other related caterogies such as hormone signalling, transcription factors etc., with a putative role in biotic stress. This finding was substantiated by further analysis using GO slim classification. It mapped 310 genes, out of which 101 genes (32.6%) belonged to the stress responsive category, the largest among the ‘known’ processes (Fig. 2b), even though many more genes are categorized under ‘unknown’ or ‘other’ processes. Singular enrichment analysis (SEA) using AgriGO, revealed the very high significance levels of the gene clusters (S3 Table). Enriched GO classification revealed that all the classes can be broadly grouped into only four categories, i.e. response to stimulus (mainly stress), protein modification, response to hormones and response to nutrient levels or starvation (Fig. 5). The largest enriched class was that of response to stimulus with 75 genes, which included several stress-related classes like defense response, response to stress, defense response-incompatible interaction and response to stimulus as the most significant classes in their decreasing order of significance level. In terms of the number of genes per cluster, the largest clusters belong to plant defensins (PDF), mildew resistance locus O proteins (MLO), and the Toll Interleukin1 receptor-nucleotide binding site-leucine-rich repeat type R (TIR-NBS-LRR) class, which are involved in biotic stress response. In addition, there are genes involved in abiotic stress such as, *Arabidopsis* Zinc Finger (AZF) and *Arabidopsis* thaliana Phloem Protein 2 A5 (ATPP2-A5). Several peroxidases and transcription factors involved in biotic and abiotic stress response were also found to be differentially expressed in the mutant.

### Hormone biosynthesis/response genes affected in GCR1 mutant

AgriGO analysis also revealed that some of the DEGs in the GCR1 mutant are involved in hormone biosynthesis as well as in response to various hormones (S3 Table). They include cytokinin oxidase/dehyrogenase (CKX4) which is involved in cytokinin biosynthesis as well in abiotic stress response, genes involved in salicylic acid response (PDF1.2, PDR12, WRKY18, GLIP1, etc), ethylene response (ERS2, ERF13, PDR12, etc) and ABA mediated pathway (LECRKA4.2, LECRKA4.3). These results indicate the prominent role of GCR1 in hormone biosynthesis/response.

### Phosphate starvation response genes among GCR1-regulated genes

Interestingly, enriched GO analysis revealed several genes related to phosphate response/starvation as downregulated in the GCR1 mutant (Table 2, Fig. 5c). They include SPX domain containing genes (SPX1, SPX3) and galactolipid/sulfolipid biosynthesis genes (MGD2, MGDC, SQD2), which are known to get differentially regulated on phosphate starvation. This finding would need further investigation.

**Table 2:** List of changed pathways with the genes and their log2fold change value involved in the pathway.

Pathway name	p-value	Genes involved	Log2fold change value
**fatty acid a-oxidation**	0.018437	AT3G01420	1.50
**sulfolipid biosynthesis**	0.018437	AT5G01220	-1.10
**camalexin biosynthesis**	0.027535	AT3G26830	-1.03
**leucopelargonidin and leucocyanidin biosynthesis**	0.0322	AT5G20550	1.15
		AT2G36690	1.12
**leucodelphinidin biosynthesis**	0.0322	AT5G20550	1.15
		AT2G36690	1.12
**flavonoid biosynthesis**	0.038214	AT5G20550	1.15
		AT2G36690	1.12
**homogalacturonan degradation**	0.038723	AT2G41850	1.83
		AT4G02330	1.11
		AT1G05310	-1.29
**glycolipid biosynthesis**	0.04549	AT2G11810	-1.61
		AT5G20410	-1.01
monolignolglucosides biosynthesis	0.05435	AT5G66690	-1.66
coniferin metabolism	0.05435	AT5G66690	-1.66
cytokinins degradation	0.063132	AT4G29740	1.74
13-LOX and 13-HPL pathway	0.063132	AT1G72520	1.22
abscisic acid biosynthesis	0.089016	AT4G18350	1.24
flavonol biosynthesis	0.154768	AT3G49620	-1.19
jasmonic acid biosynthesis	0.170489	AT1G72520	1.22
very long chain fatty acid biosynthesis	0.178245	AT5G43760	1.74
triacylglycerol degradation	0.208588	AT1G30370	1.83
IAA biosynthesis I	0.216007	AT2G30770	-1.78
ascorbate glutathione cycle	0.286654	AT3G09940	1.17
Phospholipases	0.30004	AT4G37070	1.46
superpathway of flavones and derivatives biosynthesis	0.44851	AT3G49620	-1.19

Pathways marked in **bold** are significantly changed with p-value ≤ 0.05.

### Protein kinases/phosphatases and transcription factors regulated by GCR1

**Fig 6:**
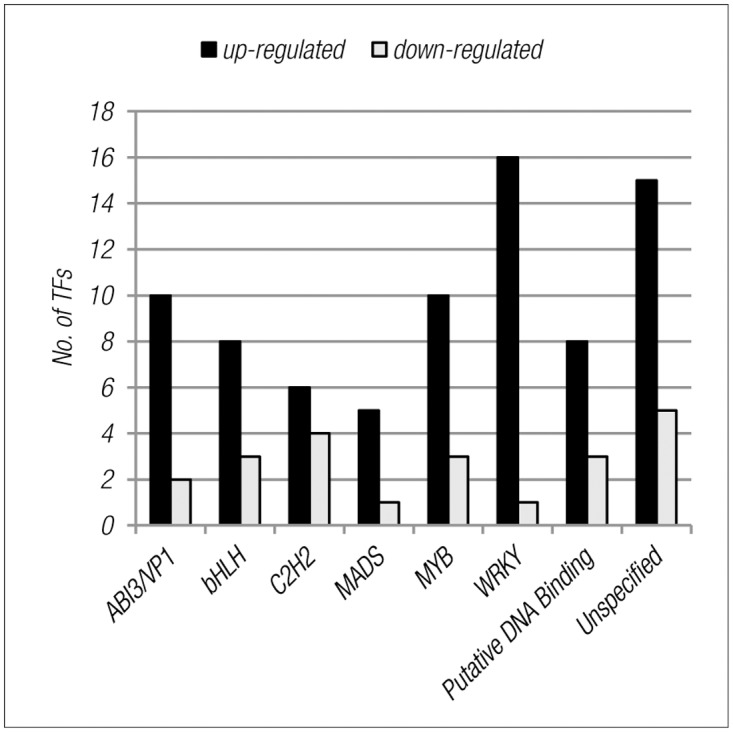
Distribution of differentially regulated transcription factors into highly represented transcription factor families.

Functional classification of DEGs identified by enriched GO analysis revealed many genes involved in protein modification by phosphorylation/dephosphorylation (S3 Table, Fig. 5d). The list included several serine/threonine kinases, leucine-rich kinases and S-locus pectin kinase families, and phosphatases like PP2C and PPCK, some of which are also annotated under stress response. Transcription and gene regulation also figured as major categories in functional classification. Comparing them with the Plant transcription factor database (PlantTFDB 2.0) revealed more than 25 transcription factor families, most of which were up-regulated in the mutant. The most represented transcription factor families were WRKY, MYB, AVI3/VP1, bHLH and C2H2 (Fig. 6). A large number of putative DNA binding and unspecified TFs also figured in the list.

### Secondary metabolite biosynthesis among GCR1-regulated pathways

When the DEG list was mapped to AraCyc [33] using plant MetGenMAP [34], most of the pathways belonged to biosynthesis of secondary metabolites. These included pathways like sulpholipid biosynthesis, fatty acid α oxidation, camalexin biosynthesis, leucopelargonidin and leucocyanidin biosynthesis, leucodelphinidin biosynthesis, flavonoid biosynthesis, homogalacturonan degradation and glycolipid biosynthesis as significantly changed pathways (p-value ≤0.05) (Table 2). Out of the above pathways, leucopelargonindin and leucocyanidin biosynthesis, leucodelphinidin biosynthesis and flavonoid biosynthesis are part of the superpathway of flavones and derivatives biosynthesis. Both the genes encoding flavanone 3-beta hydroxylase (AT5G20550, AT2G36690) were found to be up-regulated, whereas two genes involved in glycolipid biosynthesis (AT2G11810, AT5G20410) and one each in sulpholipid biosynthesis (AT5G01220) and camalexin biosynthesis (AT3G26830) were down-regulated. The DEGs involved in all other pathways were found to be up-regulated.

## Discussion

The unsettled controversy over the existence or the need for G-protein coupled receptors (GPCRs) in plant G-protein signalling has overshadowed a more fundamental quest for the overall role of GCR1 in *Arabidopsis*. We approached it from the gene discovery perspective, using functional genomics to investigate the genomewide role of GCR1 in *Arabidopsis*, including, but not limited to its more debated role in G-protein-regulated processes. For this purpose, we isolated a T-DNA knock-out mutant of GCR1 (Fig. 1, S1 Fig.) disrupted in its 2^nd^ intron, which turned out to be similar, though not identical to *gcr1–3* reported earlier [9] and also phenotypically similar to all other known GCR1 mutants [8]. It must be noted that the slightly longer, narrower leaves of this GCR1 mutant is at variance with the rounder leaves characteristic of GPA1 and AGB1 mutants that represent G-alpha and beta subunits respectively [2,35,36].

Microarray analysis of the whole plant transcriptomes of the WT and the GCR1 mutant revealed that a total of 350 genes were differentially regulated in the mutant when compared to the wild type (Fig. 2), using a stringent cut-off value of 1.0 (geometric mean log_2_) with a p-value of ≤ 0.05. These genes span all five chromosomes, indicating an extensive genomewide role for GCR1. A few of the upregulated and downregulated genes have been validated by RT-qPCR (Fig. 3, S2 Table) and the top 20 up/down-regulated genes are shown in Table 1. GOslim classification of differentially expressed genes (DEGs) revealed that over 50% of them are involved in metabolic processes, while 32.5% are responsive to stress, 19% to biotic and abiotic stimuli, 16.5% involved in transport and 15.4% in transcription, with several genes figuring in more than one category. A closer examination revealed that some of the GCR1-responsive genes/processes have close parallels to those attributed to G-protein signalling in *Arabidopsis*, whereas others have either novel roles or hither to unknown in the context of G-protein signalling, as detailed below.

### Novel roles of GCR1 similar to those attributed to G-protein signalling

Stress emerged as one of the major categories and enriched GO as well as Mapman analyses revealed that most of these differentially regulated genes pertained to biotic stress, a novel finding hitherto unknown in relation to GCR1 or any other predicted GPCR. They include genes like PDF1.2 and MLO12, which are known to be involved in innate immune response and defense against pathogens [37,38]. We also found TIR-NBS-LRR class of proteins, which have been implicated in pathogen sensing and defense response in plants [39], as well as many receptor like kinases known to be involved in plant defense signalling. Our findings on the role of GCR1 in biotic stress in this study have some close parallels with reports that implicate heterotrimeric G-proteins in biotic stress response [40,41] and plant defense signalling [42–44]. One of the reports has shown that GCR1 partners with GPA1 in the regulation of root growth mediated by bacterial quorum sensing signals [45]. At least two genes identified as GCR1 responsive in our study (PDF1.2 and PAD3), have also been reported earlier in G-protein-mediated response to biotic stress and jasmonic acid [46,47]. The role of GCR1 in abiotic stress response is by no means insignificant, even though fewer genes figure in this category than in biotic stress. Our transcriptome data corroborate the role of GCR1 and/or G-proteins in several abiotic stresses reported in separate studies, such as drought [9], heat, cold, salt [48,49], oxidative stress [50].

Secondary metabolic pathways such as camalexin and flavonoid biosynthesis constitute another novel category of GCR1-responsive processes that was previously unknown in relation to plant GPCRs. In fungi, G-protein alpha subunit was recently shown to be involved in secondary metabolism/biosynthesis of secondary metabolites [51]. Therefore, it remains to be seen whether plant heterotrimeric G-proteins mediate biosynthesis of secondary metabolites and whether they partner with GCR1.

GCR1 is involved in hormonal responses, as is evident from the categorization of the DEGs. They are involved in the biosynthesis and mediation of multiple hormonal responses like cytokinin, ethylene, salicylic acid, ABA, etc. Some of them, including ABC transporter (PDR12) and transcription factors (WRKY18) mediate hormone responsive effects that are also involved in the regulation of stress response. Heterotrimeric G-proteins in general and GPA1 and GCR1 in particular have been implicated earlier in a variety of hormone responses [8,9,52,53]. Our transcriptome data not only corroborate them category-wise, but also in terms of some of the genes involved in these processes. At least 20 of them were earlier reported in G-protein-mediated ABA response [54].

G-proteins are known to be involved in the regulation of guard cell functions and root hair differentiation [9,52], as are transcription factor complexes comprised of bHLH, MYB and WD40 domains [55]. Our finding that bHLH and MYB are GCR1 responsive raises the possibility that these TF complexes mediate the regulation of guard cell and root hair differentiation via GCR1 and G-proteins, which needs experimental validation.

Further evidence regarding the upstream GCR1 ligand and downstream GEF activity in all the above processes would be needed before it can be conclusively established that GCR1 and GPA1 act together in those processes/responses in *Arabidopsis*.

### Novel roles of GCR1 unknown or unrelated to plant G-protein signalling

MYB and WRKY class of transcription factors are known to be involved in metabolic regulation, stress and cell fate [56,57]. Our data show that the above transcription factors, as well as the above processes they regulate are GCR1 responsive, is a significant and novel observation. This is because, barring stress discussed above, the role of GPCR or G-protein has not been reported in any of them. This is also true of GCR1-regulation of AP2/EREB class of TFs found in our study, which are known to be involved in storage compound and fatty acid biosynthesis [58]. If confirmed using other G-protein mutants, it could mean that GCR1-regulation of these genes/processes works independent of GPCR/G-protein signalling.

Another novel finding of this study is that GCR1 downregulates the genes involved in response to phosphate starvation. These genes include SPX domain containing proteins (SPX1 and SPX3) and IPS1 (induced by phosphate starvation1) [59]; MGD2 and MGDC, which are involved in galactolipid metabolism during phosphate starvation [60]. Till date, no study has implicated phosphate starvation to any component of G-proteins.

## Conclusions

Our transcriptome analysis shows for the first time that a) GCR1 has an extensive genomewide response, revealing many hitherto unknown genes and processes including, but not limited to those regulated by the known G-proteins in *Arabidopsis*; b) some of its roles, such as in biotic and abiotic stress, hormone response and secondary metabolism are among those regulated by the known heterotrimeric G-proteins; and c) GCR1 also has other important roles that are either independent of, or hitherto unattributed to G-protein signalling, such as in phosphate starvation, storage compound and fatty acid biosynthesis, cell fate etc., though the possibility of some of them being regulated by other yet-to-be identified G-proteins or their components cannot be ruled out. Overall, our results point to a serious need to revisit the role of GCR1 in G-protein signalling in *Arabidopsis*, including its possible role as a GPCR. Further, considering that GCR1 and *Arabidopsis* became the main basis for contesting the existence and the role of GPCRs in plants, our results also suggest that it may be too early to write-off plant GPCRs, or their role in plant G-protein signaling.

## Supporting Information

S1 Fig Phenotypic characterization of the GCR1 mutant. (EPS)

S1 Table Correlation coefficients of microarray spot intensities across biological replicates of the wild type, Ws2 and the GCR1 mutant. (DOC)

S2 Table List of genes used for validation of microarray data, with their primer sequences and efficiencies. (DOC)

S3 Table Singular enrichment analysis (SEA) using AgriGO to obtain list of all GO biological processes with their significance levels. (DOC)
