# Correction: Transcriptome Analysis of *Arabidopsis* GCR1 Mutant Reveals Its Roles in Stress, Hormones, Secondary Metabolism and Phosphate Starvation

**DOI:** 10.1371/journal.pone.0122423

**Published:** 2015-03-19

**Authors:** The PLOS ONE Staff

The sixth author’s name is incorrect. The correct name is: Richard Hooley. The correct citation is: Chakraborty N, Sharma P, Kanyuka K, Pathak RR, Choudhury D, Hooley R, et al. (2015) Transcriptome Analysis of *Arabidopsis* GCR1 Mutant Reveals Its Roles in Stress, Hormones, Secondary Metabolism and Phosphate Starvation. PLoS ONE 10(2): e0117819. doi:10.1371/journal.pone.0117819
