# Supplementary material for: Transcriptome Analysis of Arabidopsis GCR1 Mutant Reveals Its Roles in Stress, Hormones, Secondary Metabolism and Phosphate Starvation
Source: PLoS One. 2015 Feb 10;10(2):e0117819. doi: 10.1371/journal.pone.0117819 (PMC4357605; doi:10.1371/journal.pone.0117819)
Supplement: S1 Table — (DOC) [file pone.0117819.s002.doc]

Table S1. Correlation coefficients of microarray spot intensities across biological replicates of the wild type, Ws2 and the GCR1 mutant. The data is highly reproducible as the replicates have a high correlation coefficient (~ 0.9).

| Array Name | **GCR1 mutant rep1** | **GCR1 mutant rep2** | ***ws2* rep1** | ***ws2* rep2** |
| --- | --- | --- | --- | --- |
| **GCR1 mutant rep1** | 1 | 0.980906 | 0.959246 | 0.976177 |
| **GCR1 mutant rep2** | 0.980906 | 1 | 0.973422 | 0.991414 |
| **Ws2 rep1** | 0.959246 | 0.973422 | 1 | 0.97569 |
| **Ws2 rep2** | 0.976177 | 0.991414 | 0.97569 | 1 |
