# Supplementary material for: Transcriptome Analysis of Arabidopsis GCR1 Mutant Reveals Its Roles in Stress, Hormones, Secondary Metabolism and Phosphate Starvation
Source: PLoS One. 2015 Feb 10;10(2):e0117819. doi: 10.1371/journal.pone.0117819 (PMC4357605; doi:10.1371/journal.pone.0117819)
Supplement: S2 Table — (DOC) [file pone.0117819.s003.doc]

Table S2. List of genes used for validation of microarray data, with their primer sequences and efficiencies.

| **Gene name** | **Locus id** | **Forward primer** | **Reverse primer** | **Efficiency (%)** |
| --- | --- | --- | --- | --- |
| AT4G01350 | AT4G01350 | CACCATCAACATCCTCTCCTG | CATCGCACAAGTCAAATCCAC | 100.0 |
| AT1G49570 | AT1G49570 | GTTTCATGCGCTGACATAGTTG | CAAACGGAGATGGCAGATTTG | 108.9 |
| AT2G35710 | AT2G35710 | GCAGCCTACATCTTCCCATTC | AAGCCATCAAGTAGAGCGAC | 110.0 |
| AZG2 | AT5G50300 | CCAATATCTTAGCCGACTCCG | ATTGTTCGTAGCCAGGGTTAG | 109.0 |
| PDR12 | AT1G15520 | GCGGCTTTAGGAGTCGATTT | GCTGGCTTAGCTCCTTGATTAG | 106.6 |
| AT2G02160 | AT2G02160 | CGTCTCCTGGGTTTGATGTT | TTCCCTCTCTCCTGACTTCTAC | 100.0 |
| PDF1.2 | AT5G44420 | AATGGTGGAAGCACAGAAG | TGGCTCCTTCAAGGTTAATG | 104.5 |
| GLIP1 | AT5G40990 | CCTTCACGACTATCACACTTC | CCACACGTATTGATTCCTCTC | 100.8 |
| AT5G20550 | AT5G20550 | CTTCACTCGGCACTCTCTA | GCACAGAACTCCTTGGTTAG | 98.2 |
| SPX1 | AT5G20150 | ATCTTCCCTGCTAACGAAAC | CAGCGATTGTGCTCTTCA | 97.8 |
| PAD3 | AT3G26830 | CATCCAACAACTCCACTCTT | CACGACCCATCGCATAAA | 93.0 |
| IPS1 | AT3G09922 | GACTGCAGAAGGCTGATTC | GAAGCTTGCCAAAGGATAGA | 102.1 |
| SPX3 | AT2G45130 | GCGGGAGAAGGGATATTTAG | TATTTAGCGGCGGAAGTG | 99.8 |
| AT2G36690 | AT2G36690 | GCGTGCTACATAGGGTATTG | TTTCGGCGATGGCTTTAC | 95.5 |
| FMO1 | AT1G19250 | GCGGTGTCATGCCTTTAT | CGTAGCTCTGATGTGTGTAAG | 94.4 |
| PP2-A5 | AT1G65390 | CGCTTCCTCTTCATGGACTATAC | ATGCGAGCGAGACACTAAAG | 100.3 |
| ERF13 | AT2G44840 | ACGATAACTGGAGCGACTTG | GCATCACGGAGAGTGTTGTA | 93.0 |
