# Supplementary material for: Transcriptome Analysis of Arabidopsis GCR1 Mutant Reveals Its Roles in Stress, Hormones, Secondary Metabolism and Phosphate Starvation
Source: PLoS One. 2015 Feb 10;10(2):e0117819. doi: 10.1371/journal.pone.0117819 (PMC4357605; doi:10.1371/journal.pone.0117819)
Supplement: S3 Table — (DOC) [file pone.0117819.s004.doc]

**Table S3. Singular enrichment analysis (SEA) using AgriGO to obtain list of all GO biological processes with their significance levels.**

| **GO term** | **Ontology** | **Description** | **Number in input list** | **Number in BG/Ref** | **p-value** | **FDR** |
| --- | --- | --- | --- | --- | --- | --- |
| GO:0050896 | P | response to stimulus | [75](http://bioinfo.cau.edu.cn/agriGO/termDetail.php?session=487200259&GO=GO:0050896) | 985 | 7.8e-40 | 5.4e-37 |
| GO:0006950 | P | response to stress | [51](http://bioinfo.cau.edu.cn/agriGO/termDetail.php?session=487200259&GO=GO:0006950) | 456 | 2.9e-34 | 1e-31 |
| GO:0065007 | P | biological regulation | [61](http://bioinfo.cau.edu.cn/agriGO/termDetail.php?session=487200259&GO=GO:0065007) | 882 | 4.3e-30 | 9.8e-28 |
| GO:0050794 | P | regulation of cellular process | [53](http://bioinfo.cau.edu.cn/agriGO/termDetail.php?session=487200259&GO=GO:0050794) | 658 | 4.2e-29 | 7.2e-27 |
| GO:0050789 | P | regulation of biological process | [56](http://bioinfo.cau.edu.cn/agriGO/termDetail.php?session=487200259&GO=GO:0050789) | 767 | 9.9e-29 | 1.4e-26 |
| GO:0043170 | P | macromolecule metabolic process | [61](http://bioinfo.cau.edu.cn/agriGO/termDetail.php?session=487200259&GO=GO:0043170) | 983 | 1.1e-27 | 1.2e-25 |
| GO:0043687 | P | post-translational protein modification | [21](http://bioinfo.cau.edu.cn/agriGO/termDetail.php?session=487200259&GO=GO:0043687) | 36 | 2.8e-27 | 2.7e-25 |
| GO:0042221 | P | response to chemical stimulus | [42](http://bioinfo.cau.edu.cn/agriGO/termDetail.php?session=487200259&GO=GO:0042221) | 439 | 7.7e-26 | 6.6e-24 |
| GO:0044260 | P | cellular macromolecule metabolic process | [56](http://bioinfo.cau.edu.cn/agriGO/termDetail.php?session=487200259&GO=GO:0044260) | 888 | 9.4e-26 | 7.2e-24 |
| GO:0006952 | P | defense response | [24](http://bioinfo.cau.edu.cn/agriGO/termDetail.php?session=487200259&GO=GO:0006952) | 82 | 3.2e-25 | 2.2e-23 |
| GO:0010468 | P | regulation of gene expression | [32](http://bioinfo.cau.edu.cn/agriGO/termDetail.php?session=487200259&GO=GO:0010468) | 275 | 3.1e-22 | 1.9e-20 |
| GO:0045449 | P | regulation of transcription | [30](http://bioinfo.cau.edu.cn/agriGO/termDetail.php?session=487200259&GO=GO:0045449) | 231 | 3.8e-22 | 2.2e-20 |
| GO:0019222 | P | regulation of metabolic process | [33](http://bioinfo.cau.edu.cn/agriGO/termDetail.php?session=487200259&GO=GO:0019222) | 309 | 7.7e-22 | 3.7e-20 |
| GO:0060255 | P | regulation of macromolecule metabolic process | [32](http://bioinfo.cau.edu.cn/agriGO/termDetail.php?session=487200259&GO=GO:0060255) | 284 | 7.5e-22 | 3.7e-20 |
| GO:0010556 | P | regulation of macromolecule biosynthetic process | [30](http://bioinfo.cau.edu.cn/agriGO/termDetail.php?session=487200259&GO=GO:0010556) | 238 | 8.2e-22 | 3.7e-20 |
| GO:0006350 | P | transcription | [31](http://bioinfo.cau.edu.cn/agriGO/termDetail.php?session=487200259&GO=GO:0006350) | 262 | 9e-22 | 3.7e-20 |
| GO:0019219 | P | regulation of nucleobase, nucleoside, nucleotide and nucleic acid metabolic process | [30](http://bioinfo.cau.edu.cn/agriGO/termDetail.php?session=487200259&GO=GO:0019219) | 239 | 9.1e-22 | 3.7e-20 |
| GO:0051171 | P | regulation of nitrogen compound metabolic process | [30](http://bioinfo.cau.edu.cn/agriGO/termDetail.php?session=487200259&GO=GO:0051171) | 244 | 1.6e-21 | 6e-20 |
| GO:0031323 | P | regulation of cellular metabolic process | [31](http://bioinfo.cau.edu.cn/agriGO/termDetail.php?session=487200259&GO=GO:0031323) | 268 | 1.7e-21 | 6e-20 |
| GO:0009889 | P | regulation of biosynthetic process | [30](http://bioinfo.cau.edu.cn/agriGO/termDetail.php?session=487200259&GO=GO:0009889) | 248 | 2.4e-21 | 7.8e-20 |
| GO:0031326 | P | regulation of cellular biosynthetic process | [30](http://bioinfo.cau.edu.cn/agriGO/termDetail.php?session=487200259&GO=GO:0031326) | 248 | 2.4e-21 | 7.8e-20 |
| GO:0080090 | P | regulation of primary metabolic process | [30](http://bioinfo.cau.edu.cn/agriGO/termDetail.php?session=487200259&GO=GO:0080090) | 256 | 5.4e-21 | 1.7e-19 |
| GO:0009058 | P | biosynthetic process | [46](http://bioinfo.cau.edu.cn/agriGO/termDetail.php?session=487200259&GO=GO:0009058) | 834 | 4.8e-19 | 1.4e-17 |
| GO:0010033 | P | response to organic substance | [26](http://bioinfo.cau.edu.cn/agriGO/termDetail.php?session=487200259&GO=GO:0010033) | 210 | 7e-19 | 2e-17 |
| GO:0044249 | P | cellular biosynthetic process | [43](http://bioinfo.cau.edu.cn/agriGO/termDetail.php?session=487200259&GO=GO:0044249) | 760 | 3.1e-18 | 8.6e-17 |
| GO:0006464 | P | protein modification process | [21](http://bioinfo.cau.edu.cn/agriGO/termDetail.php?session=487200259&GO=GO:0006464) | 144 | 8.9e-17 | 2.4e-15 |
| GO:0006807 | P | nitrogen compound metabolic process | [39](http://bioinfo.cau.edu.cn/agriGO/termDetail.php?session=487200259&GO=GO:0006807) | 702 | 2.3e-16 | 5.9e-15 |
| GO:0043412 | P | macromolecule modification | [22](http://bioinfo.cau.edu.cn/agriGO/termDetail.php?session=487200259&GO=GO:0043412) | 180 | 4.6e-16 | 1.1e-14 |
| GO:0010467 | P | gene expression | [35](http://bioinfo.cau.edu.cn/agriGO/termDetail.php?session=487200259&GO=GO:0010467) | 575 | 6.5e-16 | 1.6e-14 |
| GO:0034645 | P | cellular macromolecule biosynthetic process | [32](http://bioinfo.cau.edu.cn/agriGO/termDetail.php?session=487200259&GO=GO:0034645) | 482 | 1.3e-15 | 2.9e-14 |
| GO:0009059 | P | macromolecule biosynthetic process | [32](http://bioinfo.cau.edu.cn/agriGO/termDetail.php?session=487200259&GO=GO:0009059) | 485 | 1.5e-15 | 3.3e-14 |
| GO:0006139 | P | nucleobase, nucleoside, nucleotide and nucleic acid metabolic process | [34](http://bioinfo.cau.edu.cn/agriGO/termDetail.php?session=487200259&GO=GO:0006139) | 562 | 2.1e-15 | 4.4e-14 |
| GO:0019538 | P | protein metabolic process | [28](http://bioinfo.cau.edu.cn/agriGO/termDetail.php?session=487200259&GO=GO:0019538) | 374 | 5e-15 | 1e-13 |
| GO:0051707 | P | response to other organism | [16](http://bioinfo.cau.edu.cn/agriGO/termDetail.php?session=487200259&GO=GO:0051707) | 96 | 6.8e-14 | 1.4e-12 |
| GO:0016070 | P | RNA metabolic process | [21](http://bioinfo.cau.edu.cn/agriGO/termDetail.php?session=487200259&GO=GO:0016070) | 232 | 4.9e-13 | 9.6e-12 |
| GO:0044267 | P | cellular protein metabolic process | [23](http://bioinfo.cau.edu.cn/agriGO/termDetail.php?session=487200259&GO=GO:0044267) | 338 | 9.1e-12 | 1.7e-10 |
| GO:0009743 | P | response to carbohydrate stimulus | [12](http://bioinfo.cau.edu.cn/agriGO/termDetail.php?session=487200259&GO=GO:0009743) | 62 | 2e-11 | 3.8e-10 |
| GO:0009605 | P | response to external stimulus | [12](http://bioinfo.cau.edu.cn/agriGO/termDetail.php?session=487200259&GO=GO:0009605) | 65 | 3.3e-11 | 6e-10 |
| GO:0010200 | P | response to chitin | [11](http://bioinfo.cau.edu.cn/agriGO/termDetail.php?session=487200259&GO=GO:0010200) | 52 | 6.1e-11 | 1.1e-09 |
| GO:0009617 | P | response to bacterium | [9](http://bioinfo.cau.edu.cn/agriGO/termDetail.php?session=487200259&GO=GO:0009617) | 29 | 1.9e-10 | 3.3e-09 |
| GO:0042742 | P | defense response to bacterium | [8](http://bioinfo.cau.edu.cn/agriGO/termDetail.php?session=487200259&GO=GO:0042742) | 26 | 2.1e-09 | 3.5e-08 |
| GO:0016036 | P | cellular response to phosphate starvation | [6](http://bioinfo.cau.edu.cn/agriGO/termDetail.php?session=487200259&GO=GO:0016036) | 7 | 2.2e-09 | 3.6e-08 |
| GO:0016265 | P | death | [8](http://bioinfo.cau.edu.cn/agriGO/termDetail.php?session=487200259&GO=GO:0016265) | 28 | 3.4e-09 | 5.4e-08 |
| GO:0008219 | P | cell death | [8](http://bioinfo.cau.edu.cn/agriGO/termDetail.php?session=487200259&GO=GO:0008219) | 28 | 3.4e-09 | 5.4e-08 |
| GO:0006915 | P | apoptosis | [5](http://bioinfo.cau.edu.cn/agriGO/termDetail.php?session=487200259&GO=GO:0006915) | 6 | 5.7e-08 | 8.7e-07 |
| GO:0009751 | P | response to salicylic acid stimulus | [5](http://bioinfo.cau.edu.cn/agriGO/termDetail.php?session=487200259&GO=GO:0009751) | 7 | 9.7e-08 | 1.5e-06 |
| GO:0006979 | P | response to oxidative stress | [10](http://bioinfo.cau.edu.cn/agriGO/termDetail.php?session=487200259&GO=GO:0006979) | 94 | 1.7e-07 | 2.5e-06 |
| GO:0012501 | P | programmed cell death | [6](http://bioinfo.cau.edu.cn/agriGO/termDetail.php?session=487200259&GO=GO:0012501) | 20 | 2.6e-07 | 3.8e-06 |
| GO:0031667 | P | response to nutrient levels | [9](http://bioinfo.cau.edu.cn/agriGO/termDetail.php?session=487200259&GO=GO:0031667) | 81 | 5.1e-07 | 7.1e-06 |
| GO:0048583 | P | regulation of response to stimulus | [7](http://bioinfo.cau.edu.cn/agriGO/termDetail.php?session=487200259&GO=GO:0048583) | 40 | 6.2e-07 | 8.5e-06 |
| GO:0033554 | P | cellular response to stress | [9](http://bioinfo.cau.edu.cn/agriGO/termDetail.php?session=487200259&GO=GO:0033554) | 91 | 1.2e-06 | 1.7e-05 |
| GO:0009991 | P | response to extracellular stimulus | [9](http://bioinfo.cau.edu.cn/agriGO/termDetail.php?session=487200259&GO=GO:0009991) | 92 | 1.4e-06 | 1.8e-05 |
| GO:0031669 | P | cellular response to nutrient levels | [8](http://bioinfo.cau.edu.cn/agriGO/termDetail.php?session=487200259&GO=GO:0031669) | 68 | 1.5e-06 | 1.9e-05 |
| GO:0009723 | P | response to ethylene stimulus | [6](http://bioinfo.cau.edu.cn/agriGO/termDetail.php?session=487200259&GO=GO:0009723) | 30 | 2e-06 | 2.6e-05 |
| GO:0006955 | P | immune response | [14](http://bioinfo.cau.edu.cn/agriGO/termDetail.php?session=487200259&GO=GO:0006955) | 282 | 4e-06 | 5e-05 |
| GO:0031668 | P | cellular response to extracellular stimulus | [8](http://bioinfo.cau.edu.cn/agriGO/termDetail.php?session=487200259&GO=GO:0031668) | 79 | 4.2e-06 | 5e-05 |
| GO:0002376 | P | immune system process | [14](http://bioinfo.cau.edu.cn/agriGO/termDetail.php?session=487200259&GO=GO:0002376) | 283 | 4.1e-06 | 5e-05 |
| GO:0009267 | P | cellular response to starvation | [7](http://bioinfo.cau.edu.cn/agriGO/termDetail.php?session=487200259&GO=GO:0009267) | 60 | 7.1e-06 | 8.5e-05 |
| GO:0048518 | P | positive regulation of biological process | [6](http://bioinfo.cau.edu.cn/agriGO/termDetail.php?session=487200259&GO=GO:0048518) | 41 | 1e-05 | 0.00012 |
| GO:0045087 | P | innate immune response | [13](http://bioinfo.cau.edu.cn/agriGO/termDetail.php?session=487200259&GO=GO:0045087) | 266 | 1e-05 | 0.00012 |
| GO:0009607 | P | response to biotic stimulus | [18](http://bioinfo.cau.edu.cn/agriGO/termDetail.php?session=487200259&GO=GO:0009607) | 497 | 1.2e-05 | 0.00013 |
| GO:0042594 | P | response to starvation | [7](http://bioinfo.cau.edu.cn/agriGO/termDetail.php?session=487200259&GO=GO:0042594) | 66 | 1.3e-05 | 0.00014 |
| GO:0051704 | P | multi-organism process | [20](http://bioinfo.cau.edu.cn/agriGO/termDetail.php?session=487200259&GO=GO:0051704) | 605 | 1.4e-05 | 0.00015 |
| GO:0042545 | P | cell wall modification | [5](http://bioinfo.cau.edu.cn/agriGO/termDetail.php?session=487200259&GO=GO:0042545) | 26 | 1.8e-05 | 0.00019 |
| GO:0040007 | P | growth | [6](http://bioinfo.cau.edu.cn/agriGO/termDetail.php?session=487200259&GO=GO:0040007) | 51 | 3.1e-05 | 0.00033 |
| GO:0006725 | P | cellular aromatic compound metabolic process | [7](http://bioinfo.cau.edu.cn/agriGO/termDetail.php?session=487200259&GO=GO:0006725) | 82 | 4.6e-05 | 0.00048 |
| GO:0008361 | P | regulation of cell size | [5](http://bioinfo.cau.edu.cn/agriGO/termDetail.php?session=487200259&GO=GO:0008361) | 35 | 6.3e-05 | 0.00065 |
| GO:0032535 | P | regulation of cellular component size | [5](http://bioinfo.cau.edu.cn/agriGO/termDetail.php?session=487200259&GO=GO:0032535) | 36 | 7.1e-05 | 0.00071 |
| GO:0090066 | P | regulation of anatomical structure size | [5](http://bioinfo.cau.edu.cn/agriGO/termDetail.php?session=487200259&GO=GO:0090066) | 36 | 7.1e-05 | 0.00071 |
| GO:0009814 | P | defense response, incompatible interaction | [7](http://bioinfo.cau.edu.cn/agriGO/termDetail.php?session=487200259&GO=GO:0009814) | 95 | 0.00011 | 0.0011 |
| GO:0006631 | P | fatty acid metabolic process | [5](http://bioinfo.cau.edu.cn/agriGO/termDetail.php?session=487200259&GO=GO:0006631) | 40 | 0.00011 | 0.0011 |
| GO:0080134 | P | regulation of response to stress | [6](http://bioinfo.cau.edu.cn/agriGO/termDetail.php?session=487200259&GO=GO:0080134) | 68 | 0.00014 | 0.0013 |
| GO:0048869 | P | cellular developmental process | [6](http://bioinfo.cau.edu.cn/agriGO/termDetail.php?session=487200259&GO=GO:0048869) | 71 | 0.00017 | 0.0016 |
| GO:0032501 | P | multicellular organismal process | [16](http://bioinfo.cau.edu.cn/agriGO/termDetail.php?session=487200259&GO=GO:0032501) | 527 | 0.00026 | 0.0024 |
| GO:0007154 | P | cell communication | [12](http://bioinfo.cau.edu.cn/agriGO/termDetail.php?session=487200259&GO=GO:0007154) | 337 | 0.00039 | 0.0036 |
| GO:0032787 | P | monocarboxylic acid metabolic process | [6](http://bioinfo.cau.edu.cn/agriGO/termDetail.php?session=487200259&GO=GO:0032787) | 85 | 0.00043 | 0.0039 |
| GO:0009755 | P | hormone-mediated signaling pathway | [5](http://bioinfo.cau.edu.cn/agriGO/termDetail.php?session=487200259&GO=GO:0009755) | 57 | 0.00051 | 0.0045 |
| GO:0032870 | P | cellular response to hormone stimulus | [5](http://bioinfo.cau.edu.cn/agriGO/termDetail.php?session=487200259&GO=GO:0032870) | 57 | 0.00051 | 0.0045 |
| GO:0006468 | P | protein amino acid phosphorylation | [20](http://bioinfo.cau.edu.cn/agriGO/termDetail.php?session=487200259&GO=GO:0006468) | 798 | 0.00052 | 0.0046 |
| GO:0033036 | P | macromolecule localization | [6](http://bioinfo.cau.edu.cn/agriGO/termDetail.php?session=487200259&GO=GO:0033036) | 90 | 0.00057 | 0.0049 |
| GO:0032502 | P | developmental process | [16](http://bioinfo.cau.edu.cn/agriGO/termDetail.php?session=487200259&GO=GO:0032502) | 574 | 0.00063 | 0.0054 |
| GO:0008610 | P | lipid biosynthetic process | [6](http://bioinfo.cau.edu.cn/agriGO/termDetail.php?session=487200259&GO=GO:0008610) | 94 | 0.0007 | 0.0059 |
| GO:0070887 | P | cellular response to chemical stimulus | [5](http://bioinfo.cau.edu.cn/agriGO/termDetail.php?session=487200259&GO=GO:0070887) | 62 | 0.00073 | 0.0061 |
| GO:0009628 | P | response to abiotic stimulus | [11](http://bioinfo.cau.edu.cn/agriGO/termDetail.php?session=487200259&GO=GO:0009628) | 318 | 0.00085 | 0.007 |
| GO:0016310 | P | phosphorylation | [21](http://bioinfo.cau.edu.cn/agriGO/termDetail.php?session=487200259&GO=GO:0016310) | 897 | 0.00089 | 0.0072 |
| GO:0006796 | P | phosphate metabolic process | [22](http://bioinfo.cau.edu.cn/agriGO/termDetail.php?session=487200259&GO=GO:0006796) | 969 | 0.001 | 0.008 |
| GO:0006793 | P | phosphorus metabolic process | [22](http://bioinfo.cau.edu.cn/agriGO/termDetail.php?session=487200259&GO=GO:0006793) | 970 | 0.001 | 0.008 |
| GO:0048856 | P | anatomical structure development | [11](http://bioinfo.cau.edu.cn/agriGO/termDetail.php?session=487200259&GO=GO:0048856) | 330 | 0.0011 | 0.0089 |
| GO:0051716 | P | cellular response to stimulus | [16](http://bioinfo.cau.edu.cn/agriGO/termDetail.php?session=487200259&GO=GO:0051716) | 625 | 0.0015 | 0.012 |
| GO:0006351 | P | transcription, DNA-dependent | [19](http://bioinfo.cau.edu.cn/agriGO/termDetail.php?session=487200259&GO=GO:0006351) | 811 | 0.0015 | 0.012 |
| GO:0032774 | P | RNA biosynthetic process | [19](http://bioinfo.cau.edu.cn/agriGO/termDetail.php?session=487200259&GO=GO:0032774) | 812 | 0.0016 | 0.012 |
| GO:0006355 | P | regulation of transcription, DNA-dependent | [18](http://bioinfo.cau.edu.cn/agriGO/termDetail.php?session=487200259&GO=GO:0006355) | 770 | 0.0021 | 0.016 |
| GO:0051252 | P | regulation of RNA metabolic process | [18](http://bioinfo.cau.edu.cn/agriGO/termDetail.php?session=487200259&GO=GO:0051252) | 775 | 0.0022 | 0.016 |
| GO:0009620 | P | response to fungus | [6](http://bioinfo.cau.edu.cn/agriGO/termDetail.php?session=487200259&GO=GO:0009620) | 124 | 0.0027 | 0.02 |
| GO:0050832 | P | defense response to fungus | [5](http://bioinfo.cau.edu.cn/agriGO/termDetail.php?session=487200259&GO=GO:0050832) | 86 | 0.0029 | 0.021 |
| GO:0009653 | P | anatomical structure morphogenesis | [5](http://bioinfo.cau.edu.cn/agriGO/termDetail.php?session=487200259&GO=GO:0009653) | 90 | 0.0035 | 0.025 |
| GO:0007275 | P | multicellular organismal development | [13](http://bioinfo.cau.edu.cn/agriGO/termDetail.php?session=487200259&GO=GO:0007275) | 507 | 0.004 | 0.028 |
| GO:0051179 | P | localization | [14](http://bioinfo.cau.edu.cn/agriGO/termDetail.php?session=487200259&GO=GO:0051179) | 569 | 0.0041 | 0.029 |
| GO:0006810 | P | transport | [13](http://bioinfo.cau.edu.cn/agriGO/termDetail.php?session=487200259&GO=GO:0006810) | 540 | 0.0066 | 0.046 |
| GO:0051234 | P | establishment of localization | [13](http://bioinfo.cau.edu.cn/agriGO/termDetail.php?session=487200259&GO=GO:0051234) | 545 | 0.0071 | 0.049 |
